# 

**Published:** 1919-04-12

**Authors:** 


### Recent Official Publications.

To be obtained from H.M. Stationery Office, Imperial House, Kingsway, W.C. 2. All prices are quoted as postage free.

Scientific and Industrial Research. The Theory of Modern Optical Instruments. 13s.

National Health Insurance. Medical Research Committee. Special Report Series, No. 24. A Report of the Investigations of an Epidemic caused by Bacillus Ærtrycke. 10d. An Inquiry into the Prevalence and Ætiology of Tuberculosis among Industrial Workers, with special reference to Female Munition Workers. 1s. 8d.

Ministry of Labour. Reports upon Openings in Industry suitable for Disabled Sailors and Soldiers. The Musical Instruments Trade, 1919. 1½d.

Medical Supplement to the Daily Review of the Foreign Press. 1s. 1½d.

Midwives Act, 1902. Report on the work of the Central Midwives Board for the year ending March 31, 1918. 2d.

Memorandum on the Provisions of the Ministry of Health Bill, 1919, as to the work of Medical Research Committees. 1½d.

War Charities (Scotland) Bill. 1½d.

### MATRONS' AND SISTERS' APPOINTMENTS.

City of Manchester Corporation.—Miss Frances Mary Newham has been appointed assistant superintendent for maternity and child welfare. She was trained at Withington Hospitals, Manchester, later being staff nurse there, has been resident nurse at the Sandlebridge Schools Colony, matron under the Halifax Education Committee, and matron of the Crippled Children's Home, Marshside, Southport.

Bromsgrove Cottage Hospital.—Sister Margaret A. Coulcott has been appointed matron. She was trained at the North Staffs Infirmary, Stoke-on-Trent.

Metropolitan Nursing Association.—Miss Mercy Wilmshurst has been appointed superintendent. She was trained at the St. George-in-the-East Parish Infirmary, has been Queen's Nurse at Southborough, Kent, and assistant superintendent of the Brighton District Nursing Association. Miss Wilmshurst holds the certificates of the Central Midwives Board, and for Health Visitors and School Nurses.

Cumberland Infirmary, Carlisle.—Miss J. Brown has been appointed matron. She was trained at the Royal Infirmary, Preston, where, after three years' experience in other provincial hospitals, she became sister of the children's ward, night superintendent, and temporary assistant matron. Subsequently she became assistant matron of the Royal Berkshire Hospital, Reading. Miss Brown received her fever training at the Monsall Fever Hospital.

Chestnuts Sanatorium, Preston.—Miss Gertrude Wyrill has been appointed matron. She was trained at the St. Pancras (South) Infirmary; has been nurse at the Wensleydale Sanatorium, Aysgarth; staff nurse at the Maitland Sanatorium, Peppard Common; sister at the Meathop Home for Advanced Cases, and later at the Home for Early Cases, Grange-over-Sands; and sister-in-charge of the Sanatorium, Withernsea, Yorkshire. Miss Wyrill has also been tuberculosis nurse at Halifax.

Babies' Castle, Dr. Barnardo's Home, Hawkhurst.—Miss Lily A. Gostling has been appointed assistant matron. She was trained at the London Hospital, and has since been night sister and ward sister there.

General Hospital, Nottingham.—Miss Olive Roberts has been appointed theatre sister. She was trained at the Wolverhampton and South Staffordshire General Hospital, later being theatre sister there, and has done military nursing both at home and abroad.

Eye, Ear, and Throat Hospital, Shrewsbury.—Miss Nora Hendrick has been appointed sister. She was trained at the Bolingbroke Hospital, Wandsworth, and has nursed at the Royal Victoria Hospital, Dover.

Cornelia Hospital, Poole, Dorset.—Miss B. L. Lever has been appointed sister. She was trained at the Royal Victoria and West Hants Hospital, Boscombe, where she later took staff nurse's duties.

Hartlepool Hospital.—Miss Florence Phillipson, who has been appointed night sister, was trained at Stockton and Thornaby. She has held the appointment of staff nurse at Grange Hospital, Southport. Miss Olive Fairs has been appointed sister. She was trained at Monkwearmouth and Southwick Hospital, and has been night sister at Eaton, district nurse Ilkley, and staff nurse St. Chad's Hospital, Edgbaston.

Hornsey Isolation Hospital.—Miss I. Smythe has been appointed sister. Trained at Tonbridge Infirmary, she has held appointments as sister at the Southern Hospital (M.A.B.) and Brook Hospital (M.A.B.), and engaged in private nursing.

Royal Eye and Ear Hospital, Bradford.—Miss Mercy Wheeley has been appointed senior sister and assistant matron. Trained at the Royal Eye Hospital, Manchester, and the General Hospital, Altrincham, she has held the appointment of night sister at the Altrincham Hospital, has engaged in private nursing, and worked with the Q.A.I.N.S.R. Miss Helen Bell has been appointed sister of the children's ward and out-patients' department. She was trained at the Fever Hospital, Halifax, and General Infirmary, Leeds, and has held posts in the children's ward and isolation block at that institution.

Sherburn Hospital, Durham.—Miss Elizabeth Nelson has been appointed sister. She was trained at Dewsbury, Heald's Road, Infirmary, and has since engaged in private nursing and served with the British Red Cross for three years on home service and six months on foreign service.

Hull and East Riding Sanatorium, Withernsea.—Miss Browne has been appointed sister. She was trained at the Hull Royal Infirmary.

Royal Hospital for Sick Children, Edinburgh.—Miss Catherine Mary Taylor has been appointed sister to the boys' surgical ward, Miss Dorothy Phillips sister to the girls' surgical ward, and Miss Gertrude S. Thomas sister to the medical ward. Miss Taylor was trained at the Glasgow Children's Hospital and at the David Lewis Northern Hospital, Liverpool, where she was later sister of the children's ward. Miss Phillips was trained at the East London Hospital for Children, King's College Hospital, London, and for midwifery training at the University College Hospital, and has been theatre nurse at the Hostel of St. Luke, Fitzroy Square, London. Miss Thomas was trained at the David Lewis Northern Hospital, Liverpool, where she was later sister.

Blencathra Sanatorium, Threlkeld, Cumberland.—Miss Florence Keene has been appointed matron. She was trained at the Monsall Fever Hospital, Manchester; has been sister at the Derbyshire Royal Infirmary and at the Preston Isolation Hospital; and matron of the Penistone District Isolation Hospital and of the Winsley Sanatorium, near Bath.

Tunbridge Wells Homœopathic Hospital.—Miss Kathleen Welch Buckingham has been appointed nurse-matron. She was trained at the London Hospital, has been matron of the Cottage Hospital, Halstead, Essex, and has served in France under Queen Alexandra's Imperial Military Nursing Service Reserve. Miss Buckingham is a member of the College of Nursing.

### THE COLLEGE OF NURSING. (Limited by Guarantee.)

### NEWCASTLE-ON-TYNE CENTRE.

(Hon. Secretary, Miss F. Jones, Royal Victoria Infirmary.)

A meeting of members was held on March 22 in the Nursing Home of the Royal Victoria Infirmary, Newcastle-on-Tyne. The gathering was mostly of a social character, but some discussion took place on the coming election of members to the Council, and it was shown how the different Centres could help each other by combining to support any candidate they specially wished to carry. An experimental election was held. During and after tea there was much conversation and exchange of ideas between the various members, some of whom came from outlying districts. The next members' meeting, which will be the first annual meeting of the Centre, will be held in the Royal Victoria Infirmary on Saturday, April 26, at 3 p.m. All members of the College are cordially invited.

### SHEFFIELD CENTRE.

(Hon. Secretary, Miss Hancox, 334 Glossop Road.)

At a recent meeting of members held at the Royal Hospital it was decided to ask the Representative (Miss Smeeton), the Hon. Treasurer (Miss Davey), and the Hon. Secretary (Miss Hancox) to remain in office another twelve months. The members are taking large interest in the forthcoming elections to the Council, and are agreed that each Centre should have a representative on the Council. The Secretary will be pleased if any member visiting Sheffield will call upon her.

